# Microbial Eukaryotes in an Arctic Under-Ice Spring Bloom North of Svalbard

**DOI:** 10.3389/fmicb.2017.01099

**Published:** 2017-06-28

**Authors:** Archana R. Meshram, Anna Vader, Svein Kristiansen, Tove M. Gabrielsen

**Affiliations:** ^1^Department of Arctic Biology, University Centre in SvalbardLongyearbyen, Norway; ^2^Department of Biosciences, Centre for Ecological and Evolutionary Synthesis, University of OsloOslo, Norway; ^3^Faculty of Biosciences, Fisheries and Economics, UiT The Arctic University of NorwayTromso, Norway

**Keywords:** Arctic, under-ice spring bloom, microbial eukaryotes, 18S V4 region, pyrosequencing

## Abstract

Microbial eukaryotes can play prominent roles in the Arctic marine ecosystem, but their diversity and variability is not well known in the ice-covered ecosystems. We determined the community composition of microbial eukaryotes in an Arctic under-ice spring bloom north of Svalbard using metabarcoding of DNA and RNA from the hypervariable V4 region of 18S nrDNA. At the two stations studied, the photosynthetic biomass was dominated by protists >3 μm and was concentrated in the upper 70–80 m, above the thermocline and halocline. Hierarchical cluster analyses as well as ordination analyses showed a distinct clustering of the microbial eukaryote communities according to a combination of water mass and local environmental characteristics. While samples collected in the surface mixed layer differed distinctly between the two sites, the deeper communities collected in Atlantic Water were fairly similar despite being geographically distant. The differentiation of the microbial eukaryote communities of the upper mixed water was probably driven by local development and advection, while the lack of such differentiation in the communities of Atlantic Water reflects the homogenizing effect of water currents on microbial communities.

## Introduction

The Arctic spring bloom fuels production in the seasonally ice-covered shelves surrounding the Arctic Ocean. The sea ice and pelagic communities are dominated by microalgae such as diatoms (von Quillfeldt et al., 2009; Hodal et al., 2012) and to varying degrees colonies of the haptophyte *Phaeocystis pouchetii* (Eilertsen et al., 1981; Wassmann et al., 1999; Degerlund and Eilertsen, 2010). During the last decade, sequencing of 18S rDNA has been used to metabarcode communities of also the pico- (0.2–3 μm) and nano- (3–20 μm) sized eukaryotic fraction of Arctic marine systems (e.g., Lovejoy et al., 2006; Comeau et al., 2011; Kilias et al., 2014; Marquardt et al., 2016) as reviewed by Lovejoy (2014). The insights of arctic microbial eukaryotes gained by the use of molecular genetic tools include the identification of ecotypes endemic to the Arctic (Lovejoy et al., 2007; Terrado et al., 2013; Percopo et al., 2016), the existence of taxa-specific biogeographic patterns (Thaler et al., 2015), and a linking of distinct protist assemblages to water masses (Hamilton et al., 2008; Bachy et al., 2011; Monier et al., 2014; Metfies et al., 2016). It has been shown that the pronounced Arctic seasonality extends to the succession of microbial eukaryotes (Marquardt et al., 2016; Joli et al., 2017). Molecular data can also provide insight into responses of marine microbes to environmental change (Comeau et al., 2011) with potential changes higher up in the marine food web.

The area north of Svalbard in the European Arctic is seasonally covered by sea ice, although with a diminishing ice concentration the last decade (Onarheim et al., 2014). The region is strongly influenced by inflow of Atlantic Water (AW) with relatively high salinity and temperature (≥34.8 and 2°C, respectively) from the West Spitsbergen Current (Cokelet et al., 2008). Increased temperature in the inflowing AW (Walczowski et al., 2012) is probably the main driver of the reduced sea ice concentration in the area which is seen predominantly in winter (Onarheim et al., 2014). At the same time there is a trend of increased boreal plankton in the Atlantic inflow water (Weydmann et al., 2014; Paulsen et al., 2016) pointing to the importance of AW for the transport of taxa along the Spitsbergen coastline.

The upper mixed layer north of Svalbard is characterized by reduced salinity (≤ 34.4; Manley, 1995) due to riverine input and melting of sea ice throughout the Arctic Ocean (Lind and Ingvaldsen, 2012). Upwelling of the deeper AW to the surface was demonstrated in the winter of 2012 along the northern Svalbard shelf (Falk-Petersen et al., 2015), thus mixing this more saline and warmer water and its northward transported plankton to the surface. The extreme environmental variability in this high Arctic region makes it an excellent area to study the contrasts between homogenization of the microbial communities due to potential large-scale dispersal with water currents and environmental filtering due to local conditions.

In this study, we investigated microbial eukaryote diversity and community compositional differences in an under-ice spring bloom north of Svalbard. Our main objectives were: (1) To indentify the community of microbial eukaryotes in two ice-covered stations, (2) to unravel the influence of water masses of different characteristics and history vs. local processes on the composition of the microbial protist communities, and (3) to study the metabolically active fraction of the community by comparing inventories based on rRNA and the rRNA gene.

## Materials and methods

### Sample collection

**Figure 1:**
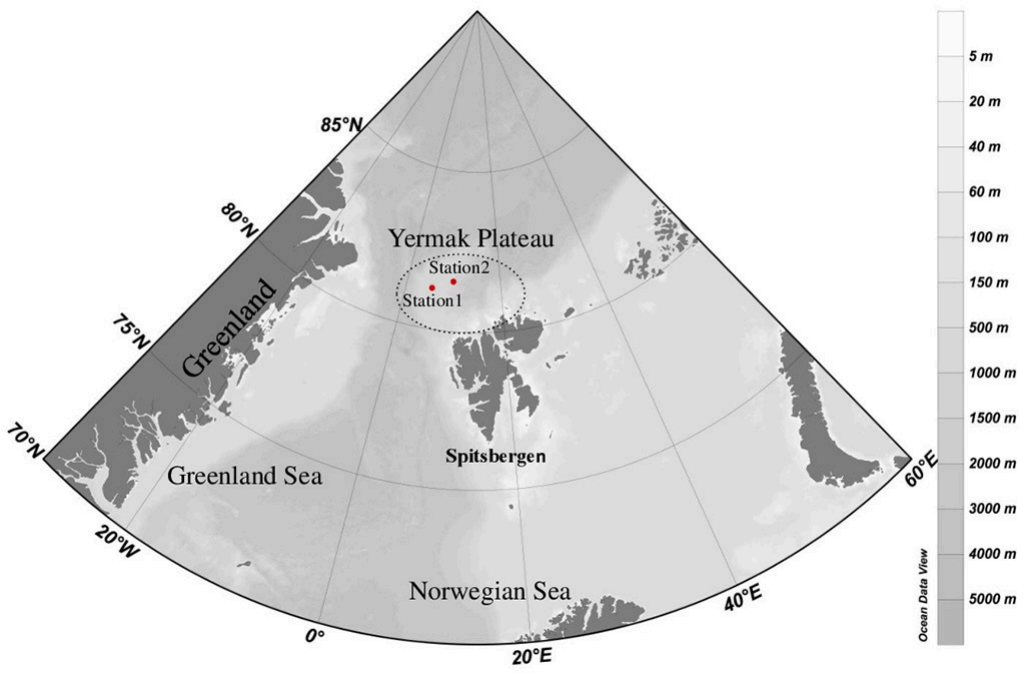
Map of the studied area showing the locations of the two sampling stations (Station 1, Station 2) NW of Spitsbergen.

Seawater samples were collected in May 2010 from two ice-covered stations, Stations 1 and 2, both located north of Spitsbergen (Figure 1). Station 1 in this paper was sampled between St. 2 and St. 3 of Johnsen et al. (submitted). After having drifted out of the sea ice, as described by Johnsen et al. (submitted), the ship re-located further NE into the sea ice, where our Station 2 was sampled. Sampling depths were selected based on hydrography and fluorescence profiles aiming to sample potentially different habitats for microbial protists. Water samples from five depths (0, 10, 45, 65, and 200 m) were collected at Station 1 and from four depths (0, 15, 120, and 200 m) at Station 2. From each depth, seawater was sequentially filtered through 3 μm filters (Isopore membrane filter, Millipore, USA) onto 0.22 μm filters (Durapore membrane hydrophilic PVDF filter, Millipore, USA) using a peristaltic pump at 40 rpm. One L was filtered for DNA extraction and 2 L were filtered for RNA extraction. Immediately after filtration, the RNA filters were submerged in 1 mL of Trizol (Invitrogen), the tube was shaken vigorously and kept at room temperature for 5 min. Filters were snap-frozen in liquid nitrogen and kept at −80°C until extraction. To measure total and >3 μm Chl *a* biomass, 250 mL seawater was filtered in triplicate onto GFF filters (Whatman, England) and 3 μm Isopore membrane polycarbonate filters (Millipore, USA), respectively. The filters were extracted in methanol for 12–24 h at 4°C in the dark and analyzed using a 10-AU-005-CE fluorometer (Turner, USA). Seawater for nutrient analyses was collected in acid-washed and sample-rinsed bottles and kept frozen until analysis. Nitrate + nitrite (NO_3_+NO_2_), phosphate (PO_4_), and silicate (Si(OH)_4_) were analyzed in triplicate by standard seawater methods using a Flow Solution IV analyzer from O.I. Analytical, USA. Photosynthetically active radiation (PAR) from the surface down to 40 m was measured using a cabled, flat Li-Cor sensor (LI-1400). The temperature, salinity and density measurements taken from the onboard SeaBird CTD was plotted in a TS diagram, and the water masses were characterized according to Aagaard and Carmark (1989); Manley (1995), and Cokelet et al. (2008). The thickness of the sea ice and the snow depths on top of the sea ice were measured in 6–10 drilled holes per station.

### Nucleic acid extraction and pyrosequencing

DNA was extracted using a modified CTAB protocol according to Vader et al. (2014) and each filter was cut into two halves and extracted individually. After thawing the filters sampled for RNA, the Trizol was transferred to a new tube and the filter was washed with 1 mL of fresh Trizol. In both tubes, the cells were lysed by beating two times 1 min with 200 μm zirconium beads (Molecular grade, pre-filled tubes, OPS Diagnostics) in a bead beater at 1/22s. Subsequently total RNA was extracted according to the manufacturers' protocol, except that 10 μg glycogen (RNA grade, Fermentas) was added to each tube as a carrier during precipitation of the RNA. The RNA was dissolved in DEPC-treated water, and DNAse-treated with Turbo DNAse (Ambion) according to the manufacturers protocol. RNA was reverse transcribed using the Retro Script kit (Ambion) with random decamers at 44°C for 1 h, after an initial denaturation step of 3 min at 75°C.

The hypervariable V4 18S rDNA region was amplified from the rRNA gene and from cDNA from reverse transcribed rRNA (referred to DNA and RNA, respectively) using eukaryote-specific 454 primers (Comeau et al., 2011). Samples were amplified in triplicate 25 μl reactions containing 1xHF-buffer, 0.2 μM of each dNTP, 0.2 μM of each primer, 0.5 U Phusion Hot start HF II polymerase and 1 μl undiluted DNA or RNA. All products were cleaned using AMPure XP beads (Agencourt, Beckman Coulter) according to the manufacturers instruction using a 4:5 ratio of beads to PCR product. Subsequently all PCR products were pooled at equimolar amounts and sequenced unidirectionally on a Roche 454 GS-FLX Titanium platform at the IBIS/Plateforme d'Analyses Génomiques de l'Université Laval.

### Sequence processing and statistical analysis

Sequence analyses were done in QIIME v1.5.0 and v1.8.0 (Caporaso et al., 2010). Low quality reads were removed using default parameters except for min quality score 30, min and max sequence lengths 330 and 550 bp, max homopolymer length 7, and no ambiguous bases. Reads that passed the quality control were denoised using DeNoiser v0.851 (Reeder and Knight, 2010) as implemented in QIIME v1.5.0. Chimeras were removed in MOTHUR v1.32.1 (Schloss et al., 2009) with uchime (Edgar et al., 2011) using self as reference and default settings. Demultiplexed reads were clustered into operational taxonomic units (OTUs) at 98% sequence similarity (as often used in protist metabarcoding; cf. Comeau et al., 2011) using UCLUST (Edgar, 2010) with default parameters and the taxonomy of the most abundant representatives from each OTU were assigned using BlastN (*e*-value 0.00001) against the curated protist databases of Lovejoy et al. (2016) and PR2 (gb203; Guillou et al., 2013). Global singletons and reads assigned to Metazoa were removed. The number of sequence reads per sample was rarefied by random sampling to the sample having the lowest read number (6066). Diversity analyses were performed using the Vegan (version 2.0–10) package in R (Version 0.98.994–RStudio, Inc.). The Shannon diversity index and Pielou's index of evenness were calculated to compare alpha-diversity between the samples. Rarefaction analyses were conducted and computed every 100 sequences using 5,000 subsampling iterations.

Beta diversities were analyzed by clustering samples using UPGMA (unweighted pairwise grouping method with averaging) based on Bray-Curtis dissimilarities. To identify a possible differentiation of the communities under the constraint of environmental factors, canonical correspondence analysis (CCA) was done using the PAST software (v3.04, Hammer et al., 2009). CCA was performed on a Bray-Curtis dissimilarity matrix of the samples. A permutation test was performed to calculate the significance of constraints on the data for the environmental parameters (fluorescence, density, depth, temperature, salinity, PAR and concentrations of nitrate/nitrite, phosphate, and silicate). The environmental parameters were tested for statistical dependence using the Spearman rank correlation coefficient (data not shown). For two highly co-varying parameters (salinity co-varied with density and silicate co-varied with phosphate) only one parameter was shown.

To visualize the differences among the libraries, a heat map of relative abundances was generated (in R) for the abundant OTUs (>1% of total reads) and the five most abundant OTUs in each library, and overlaid the UPGMA dendrogram.

## Results

### Study location and hydrography

**Figure 2:**
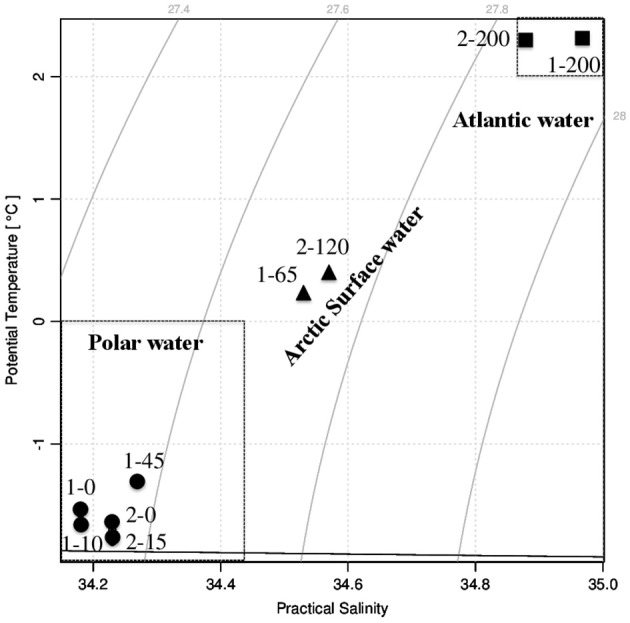
Temperature-salinity diagram showing the water masses present at sampling Stations 1 and 2. The names of the collected water samples were according to their station number and depth. Circles represent Polar Water (PW) samples, triangles represent Arctic Surface Water (ASW) samples, and squares represent Atlantic Water (AW) samples.

**Figure 3:**
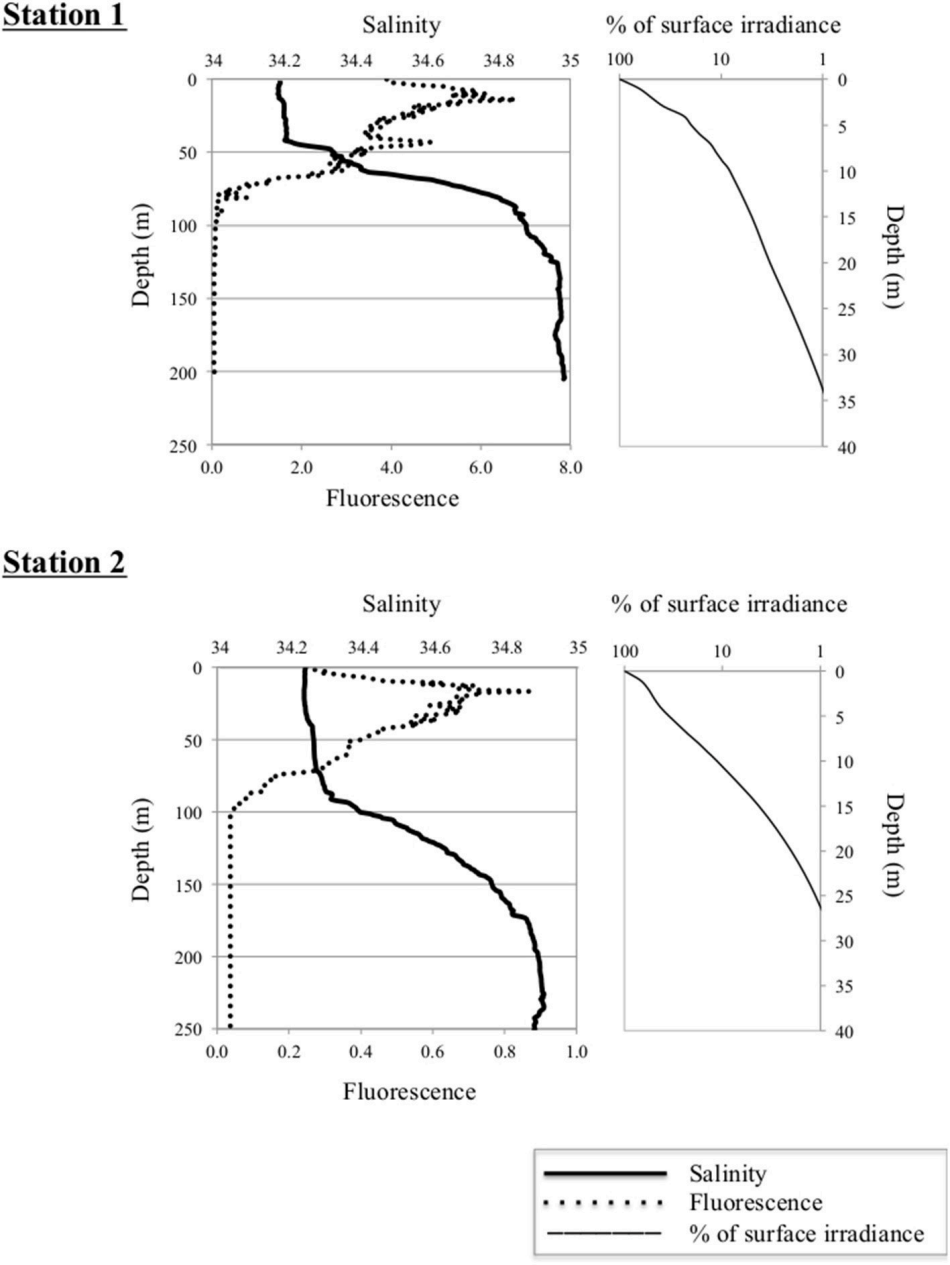
**Left panels:** Vertical distribution of salinity and *in situ* fluorescence (mg Chl *a* m^−3^) at Stations 1 **(upper panel)** and 2 **(lower panel)**. Depth is indicated on the Y-axis. **Right panels:** Percentage of surface irradiance with depth at the two stations.

At the time of sampling both ice stations were snow-covered (30 ± 9.7 cm and 23 ± 6.3 cm at Station 1 and Station 2, respectively) and had thick overlying sea ice (121 ± 11.7 cm and 160 ± 32.3 cm at Station 1 and Station 2, respectively). The water masses were identified based on their potential temperature—salinity characteristics; Atlantic Water (AW), Polar Water (PW), and Arctic Surface Water (ASW; Aagaard and Carmark, 1989; Cokelet et al., 2008; Figures 2, 3). The hydrographic profiles of the two stations were similar, although the PW was deeper at Station 2 (90 m) compared to Station 1 (45 m; Figure 3). The upper samples from both stations were all collected within the PW (S < 34.4; θ < 0°C), and designated PW1 (0, 10, and 45 m from Station 1) and PW2 (0 m and 15 m from Station 2; Table 1). The samples from 200 m at both locations displayed Atlantic Water properties. The samples from depths 65 m at Station 1 and 120 m at Station 2 were collected beneath the halocline and thermocline at the two stations, and represented Arctic Surface Water (ASW1 and ASW2 from stations 1 and 2, respectively). The two stations both consisted of ice floes with leads in between, and the euphotic zone (1% of surface PAR) reached 27 m at Station 1 and 34 m at Station 2 (Figure 3).

**Table 1:** Environmental characteristics of the water samples collected from North-West Spitsbergen.

**Station_depth**	**Temp^a^**	**Sal^b^**	**Den^c^**	**Chl *a*^d^ >3 μm**	**Chl *a* 3–0.7 μm**	**Nitrate+Nitrite^e^**	**Phosphate^e^**	**Silicate^e^**	**Origin of water**	**No. OTUs DNA/RNA**	**Shannon diversity index**	**Pielou's evenness index**
1_0 m	−1.57	34.18	27.51	15.97	bd	2.38	0.30	1.07	PW	193/114	2.63/2.12	0.50/0.45
1_10 m	−1.65	34.18	27.51	15.54	bd	2.82	0.33	1.29	PW	185/131	2.31/1.95	0.44/0.40
1_45 m	−1.31	34.27	27.62	10.70	0.39	1.27	0.23	0.60	PW	253/174	2.50/3.0	0.45/0.58
1_65 m	0.22	34.53	27.76	na	na	5.86	0.65	2.02	ASW	293/205	3.14/3.04	0.55/0.57
1_200 m	2.32	34.97	27.93	na	na	6.88	0.66	3.12	AW	439/416	4.69/3.98	0.77/0.66
2_0 m	−1.7	34.23	27.56	1.33	0.08	2.70	0.36	0.97	PW	312/157	2.97/2.19	0.52/0.43
2_15 m	−1.8	34.23	27.56	1.01	0.06	2.58	0.36	0.92	PW	157/133	2.28/2.18	0.45/0.45
2_120 m	0.39	34.57	27.74	na	na	2.05	0.49	0.59	ASW	325/390	4.21/3.94	0.73/0.66
2_200 m	2.31	34.88	27.86	na	na	3.94	0.58	1.75	AW	400/459	4.58/4.44	0.76/0.72

*Samples from Station 1 (80.40° N, 5.60° E) and Station 2 (81.23° N, 9.30° E) were sampled on 16th and 19th of May, 2010, respectively. PW, Polar Waters; ASW, Arctic Surface Waters; AW, Atlantic Waters. No. of OTUs mentioned in the table are after subsampling. The OTU numbers are written for both DNA/RNA samples, where upper numbers are from DNA samples and lower numbers are from RNA samples. The estimated diversity indices values were also written in a similar way (DNA/RNA)*.

a *Temperature °C*.

b *Salinity*.

c *Density*.

d Chl a measured in μg L^−1.^

e *μM; na: not analyzed*.

*bd, below detection level*.

A distinct algal bloom had developed at Station 1, with Chl *a* concentrations of 10.7–16.0 μg L^−1^ in the PW (Table 1). Station 2 saw an order of magnitude lower Chl *a* biomass at 1.0–1.3 μg L^−1^ in the PW. The size fractionated Chl *a*-values showed that cells >3 μm dominated the photosynthetic biomass at both stations; at Station 1 the autotrophic biomass of the picoplanktonic fraction was low or below detection, and at Station 2 it was very low (0.06–0.08 μg L^−1^).

The depth distribution of the measured nutrients (NO_3_ + NO_2_, PO_4_, Si(OH)_4_) was similar at the two stations, with comparatively low nutrient concentrations in the PW, minimum concentrations at intermediate depths (120 m at Station 2) and considerably higher concentrations in the AW (Table 1).

### Sequence analyses and diversity estimates

A total of 286,979 reads were obtained, which were reduced to 224,272 after quality filtering and chimera checking. Clustering the reads at 98% similarity produced 1,547 OTUs, which after removing OTUs classified as Metazoa, global singletons and subsampling to the smallest sample size (6,066 reads) were further reduced to 1,288 OTUs. The rarefaction curves (Figure S1) showed that all libraries had been sequenced to saturation. There was a general trend of increasing diversity with depth both in OTU richness and evenness, also reflected in the Shannon-Wiener diversity index (Table 1). A higher eukaryotic diversity was seen in the DNA samples compared to the RNA samples.

### Microbial eukaryote community structure

**Figure 4:**
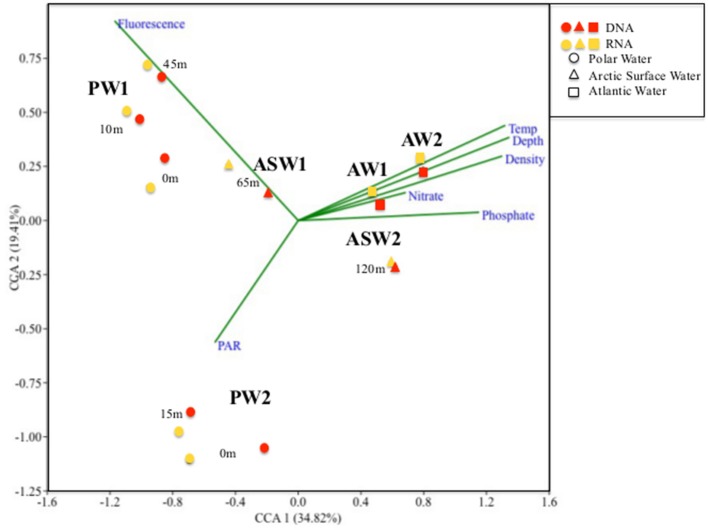
Canonical Correspondence Analyses (CCA) ordination diagram of the 18 samples collected at different depths from Stations 1 and 2. PW, Polar Water; ASW, Arctic Surface Water; and AW, Atlantic Water. 1 and 2 refers to the respective Station numbers (i.e., PW1-Polar Water samples from Station1). Arrows represent the best explanatory environmental and biological variables fitted onto the ordination space.

**Figure 5:**
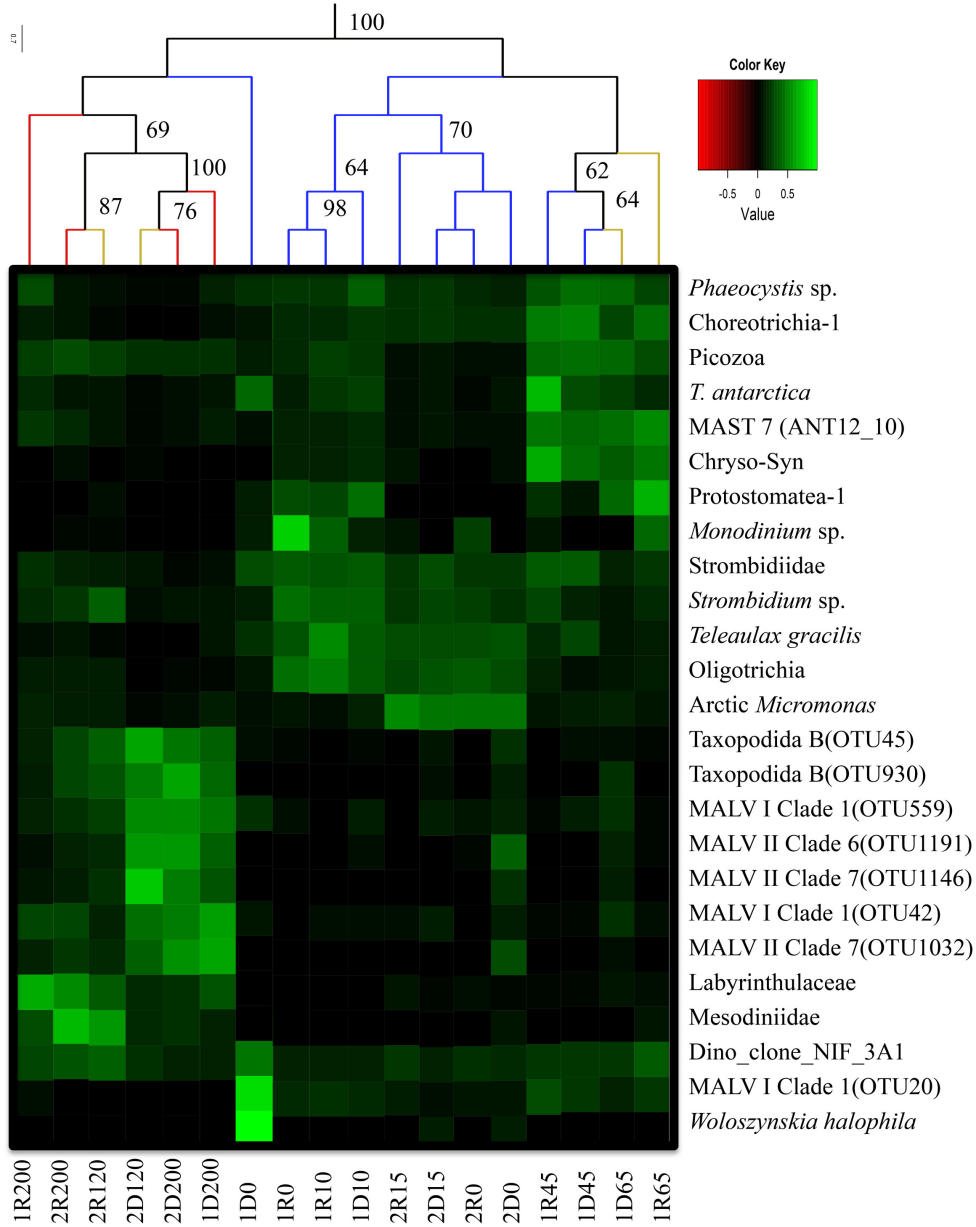
Heatmap of abundant OTUs (>1% of the reads in the total data set) and the 5 most abundant taxa from each library. Taxonomic assignments refer to Table 2. The corresponding value scale is displayed as a color gradient (green: abundant; black: rare). The column dendrogram was prepared based on all OTUs and represents neighbor joining clustering with bray-curtis distances. Bootstrap values was based on 999 permutations, and values below 60 are not shown. Colored branches in the dendrogram shows different watermasses (Red: AW; Orange: ASW; Blue: PW). Naming of samples: 1 or 2 states the sampling stations, D or R refer to the DNA or RNA samples and the number refers to sampling depth (i.e., 1R200- RNA sample from 200 m from Station 1). This is a modified image after combining the heatmap and clustering figures together to enhance the illustration.

Hierarchical cluster analyses as well as the CCA showed a distinct clustering according to a combination of water mass and local environmental characteristics (Figures 4, 5) where the two first axes in the CCA described 54.2% of the variation in the data. The two samples collected from ASW were clearly associated with the communities of either AW (the ASW2 sample from 120 m depth; Figures 4, 5) or PW (the ASW1 sample clustered with the PW1 samples, in particular with the 45 m PW1 sample; Figure 5). The permutation test performed on the CCA confirmed the importance of water mass as a structuring factor, as significant interactions were identified between the community composition of individual samples and density (*R*^2^ = 0.13, *p* = 0.01). In addition, the ANOVA identified fluorescence (*R*^2^ = 0.19, *p* = 0.001) as the other significant factor structuring the communities. While density mostly separated along the first CCA axis (which explained 34.8% of the variability in the data), fluorescence separated the communities along the second CCA axis (which explained 19.4% of the variability in the data) thus separating the surface samples of the two stations. Other parameters explaining the variability seen in the community composition (although not statistically significant) were temperature, depth, nutrient concentrations and PAR (Figure 4).

### Microbial eukaryote communities

**Figure 6:**
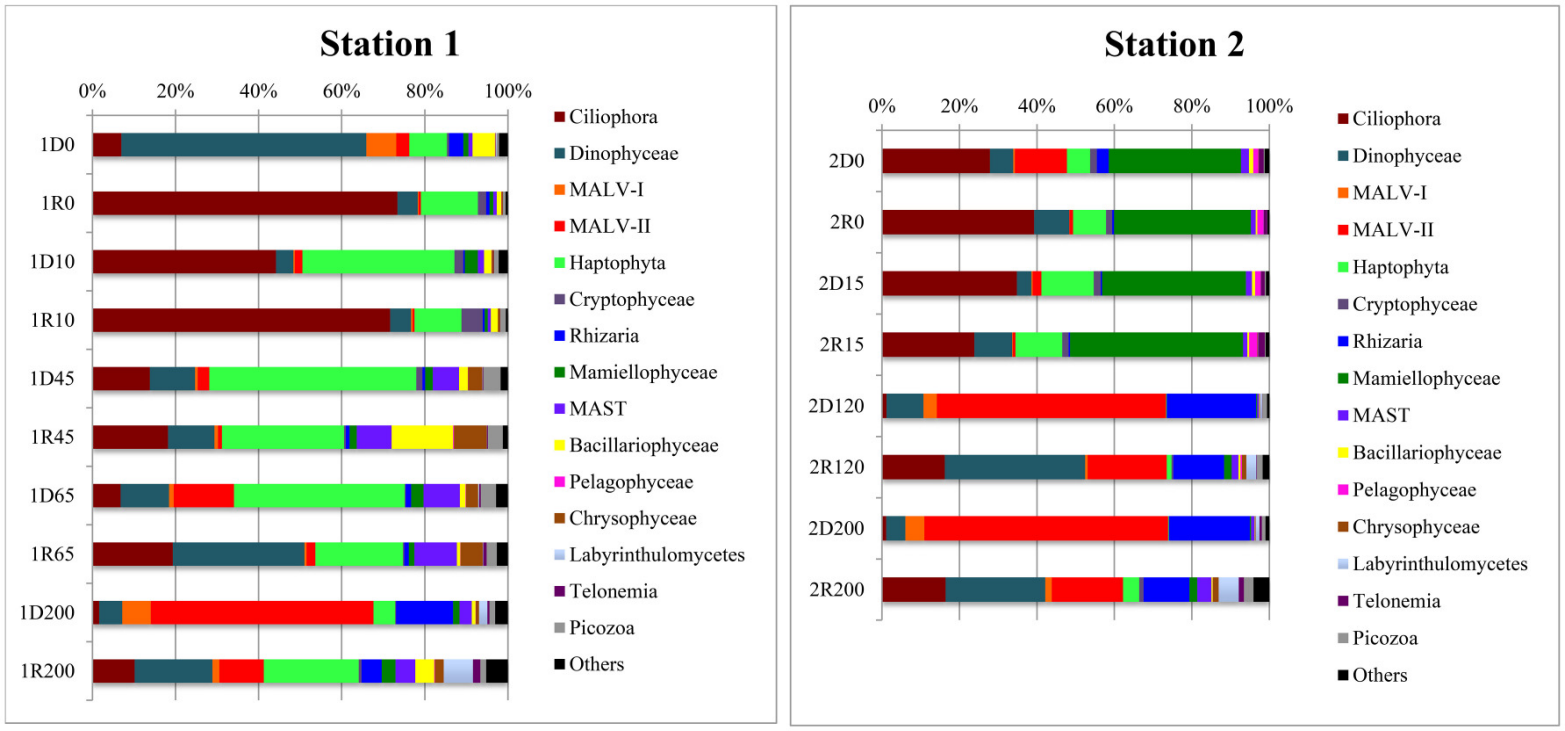
Barchart of eukaryotic OTU (98% sequence similarity) composition in DNA and RNA samples from different depths at Stations 1 and 2. Taxonomy according to Adl et al. (2012). OTUs that did not give clear taxonomic information in BLAST and that had <1% reads in every sample were lumped together in “Others.” The sample names used in this figure is similar to Figure 5.

Alveolates were abundant in all samples, recruiting 30–80% of reads in individual libraries (Figure 6). While Ciliophora dominated in PW (7–73% of library reads), Dinophyceae and MALV were more common in AW (including the 120 m ASW sample from Station 2). The Dinophyceae reads were dominated by one abundant OTU (assigned to Dino_clone_NIF_3A1; Table 2, Figure 5) that accounted for 3–44% of the total reads in individual libraries and was present in all libraries. While the maximum number of reads was detected in the 0 m DNA sample from Station 1 (1D0 library), RNA abundances were highest in the deep samples (all AW and 65 m from Station 1, Figure 5). Reads assigned to *Woloszynskia halophila* was also highly abundant in the 1D0 library, but did not have a similar read abundance in the RNA library (1R0; Figure 5). Reads assigned to MALV were more abundant in the DNA libraries than in the RNA libraries, while the dominant Ciliophora and Dinophyceae OTUs displayed the opposite pattern, recruiting a larger proportion of reads within the RNA libraries (Figure 5).

**Table 2:** Overview of the relative abundances and assignments of the abundant OTUs (>1% of total reads and/ or the five most abundant OTUs in each library).

**Assignment (OTU name)**	**Name used in the text**	**Group**	**Accession no.**	**% Similarity**	**% of total reads across all libraries**	**Database**
Phaeocystis_sp.	*Phaeocystis* sp.	Haptophyta	KC488454	99	15.8	PR2
Oligotrichia_XX_sp.	Oligotrichia	Ciliophora	KC488393	99	15.4	PR2
Dino_clone_NIF_3A1	Dino_clone_NIF_3A1	Dinophyceae	EF526808	100	11.5	CLJ
Micromonas_CCMP2099_Arctic	Arctic *Micromonas*	Mamiellophyceae	DQ025753	99	9.6	CLJ
Strombidiidae_X_sp.	Strombidiidae	Ciliophora	KJ762428	100	2.4	PR2
MAST_7; ANT12_10	MAST 7 (ANT12_10)	MAST	AF363196	99	1.6	CLJ
Dino-Group-II-Clade-7_X_sp.	MALV II Clade 7 (OTU1032)	MALV-II	KJ763748	99	1.2	PR2
Picobiliphyta_XXXX_sp.	Picozoa	Picozoa	KC488345	99	1.2	PR2
RAD-B-Group-IV_X_sp.	Taxopodida B (OTU45)	Rhizaria	KJ762540	99	1.2	PR2
Choreotrichia-1_X_sp.	Choreotrichia-1	Ciliophora	HM561031	99	1.1	PR2
Thalassiosira_antarctica	*Thalassiosira antarctica*	Bacillariophyceae	KC771154	99	1.1	PR2
Teleaulax_gracilis	*Teleaulax gracilis*	Cryptophyceae	JQ966994	99	1	CLJ
Strombidium_sp.	*Strombidium* sp.	Ciliophora	KJ762448	99	1	PR2
Dino-Group-I-Clade-1_X_sp.	MALV I Clade 1 (OTU559)	MALV-I	KJ761596	99	1	PR2
Monodinium_sp.	*Monodinium* sp.	Ciliophora	DQ487196	99	0.9	CLJ
Dino-Group-I-Clade-1_X_sp.	MALV I Clade 1 (OTU42)	MALV-I	KJ763264	99	0.8	PR2
Mesodiniidae_X_sp.	Mesodiniidae	Ciliophora	AY665074	99	0.7	PR2
Clade-H_X_sp.	Chryso-Syn	Chrysophyceae	HQ869056	99	0.7	PR2
Labyrinthulaceae_X_sp.	Labyrinthulaceae	Labyrinthulomycetes	HQ869249	99	0.7	PR2
Dino-Group-II-Clade-7_X_sp.	MALV II Clade 7 (OTU1146)	MALV-II	KJ762821	99	0.6	PR2
Polycystinea_Taxopodida_B	Taxopodida B (OTU930)	Rhizaria	AF382825	99	0.6	CLJ
Dino-Group-II-Clade-6_X_sp.	MALV II Clade 6 (OTU1191)	MALV-II	KJ762287	99	0.6	PR2
Dino-Group-I-Clade-1_X_xp.	MALV I Clade 1 (OTU20)	MALV-I	KJ763264	99	0.5	PR2
Woloszynskia_halophila_strain_WHTV_S1	*Woloszynskia halophila*	Dinophyceae	EF058252	100	0.4	CLJ
Protostomatea-1_XX_sp.	Protostomatea-1	Ciliophora	FN690354	99	0.4	PR2

*Assignment represents best hit to the lowest taxonomic level using blastn with e-value 0.00001 against databases PR2 (Guillou et al., 2013) and CLJ (Lovejoy et al., 2016). Assignment to CLJ was used in cases having identical % identity for both databases*.

Three Ciliophora OTUs were among the abundant taxa; Choreotrichia-1 was abundant in the 45 and 65 m libraries from Station 1, whereas Strombidiidae and Oligotrichia were prevalent in PW, with especially high read numbers of the latter in RNA from Station 1 (Figure 5). Four additional Ciliophora OTUs were among the five most abundant in single libraries; *Strombidium* sp. did not show a very distinct distribution pattern, but was most abundant in PW, *Monodinium* sp. was particularly abundant in the 0 m RNA sample of Station 1, Mesodiniidae was abundant in the RNA libraries from AW, and Protostomatea-1 was found in the PW (including at 65 m) of Station 1.

The haptophytes were dominated by an OTU assigned to *Phaeocystis* sp. (Table 2), which was the most abundant OTU in the total dataset (16% of all reads) and was especially prevalent in the PW (and ASW) at Station 1 (6–49% of library reads). The same OTU also recruited 5–21% of all reads in the deeper AW sample from the same station. Another dominant group was the pico-sized green algal phylum Mamiellophyceae which recruited 35–45% of reads from the PW samples of Station 2 (Figure 5). Nearly all of these sequences were assigned to the Arctic ecotype of *Micromonas* (Arctic Micromonas; Table 2).

A Bacillariophyceae OTU assigned to *Thalassiosira antarctica* was found at both stations, but showed highest relative abundance in PW at 45 m of Station 1. OTUs assigned to marine stramenopiles were found throughout the water column at both stations, with the most abundant OTU (MAST 7; ANT12_19) along with an OTU assigned to Chrysophyceae-Synurophyceae Clade-H predominating at 45 m and 65 m in Station 1 (Figures 5, 6). Another stramenopile OTU, assigned to Labyrinthulaceae, was only abundant in AW. An OTU assigned to the cryptophyte *Teleaulax gracilis* was abundant in PW at both stations, but had higher RNA read numbers at Station 1 (Figure 5). Picozoa were found at all depths at both stations, but were most abundant in the libraries from intermediate and deeper depths (Figure 6). An OTU assigned to the rhizarian Cryomonadida (OTU 20) was highly abundant in the surface DNA library from Station 1. Other abundant taxa in the deep AW included two OTUs assigned to the radiolarian Taxopodida B (Table 2, Figures 5, 6).

### DNA vs. RNA

The results of the UPGMA clustering indicated that among the deeper samples (120 and 200 m), libraries made from the same molecule type (DNA vs. RNA) were more similar than libraries from the same location or depth (Figure 5). In particular, reads assigned to the two Taxopodida B and the five abundant MALV OTUs were more abundant in the DNA libraries compared to the RNA libraries. This effect was, however, not apparent in the CCA analysis where libraries prepared from the same depth and station were most similar (Figure 4). A higher number of rare OTUs were identified from the DNA libraries compared to the RNA libraries, whereas the numbers of abundant OTUs were similar in the two molecule types (data not shown).

## Discussion

### Surface water community differentiation

The two stations showed limited sympagic growth underneath the melting sea ice (personal observation), and the depth of the salinity-reduced surface water (PW; 45 m at Station 1 and 90 m at Station 2; Figure 3) suggest that sea ice melting had been ongoing for a while, although typical sea ice algae as well as the dinoflagellate *Polarella glacialis* with resting cysts were identified in the bottom 3 cm of ice cores taken in the vicinity (Johnsen et al., submitted). The high Chl *a* concentrations along with reduced nutrient concentrations and the algal community composition suggest that Station 1 was in a peak bloom phase (cf. Kristiansen et al., 2000; Johnsen et al., submitted). Station 2, on the other hand, had an order of magnitude lower Chl *a* concentrations while nutrient levels were similar to those of Station 1 (Table 1). Although a permanent stratification with surface mixed layer nitrate values around 5 μM may exist in the region in the pre bloom phase (Randelhoff et al., 2016), the low Chl *a* and nutrient concentrations at Station 2 suggest that this area had already developed into a post-bloom stage. The more shallow euphotic zone at Station 1 was probably due to shading from the ongoing bloom (Table 1, Figure 3).

The pelagic bloom in Station 1 was dominated by large and typical spring-bloom species of diatoms and had probably advected into the area (Johnsen et al., submitted). Common spring bloom species of Arctic waters such as *Phaeocystis* sp. and *Thalassiosira antarctica* (von Quillfeldt, 1997; Degerlund and Eilertsen, 2010) were among the most abundant OTUs in our dataset (Figures 5, 6), and were also abundant in phytoplankton samples from the area studied by microscopy (Johnsen et al., submitted). An advection of *Phaeocystis* sp. into ice-covered polar water in the Fram Strait was suggested also by Metfies et al. (2016), although a recent study report an under-ice bloom dominated by *Phaeocystis* sp. developing *in situ* (Assmy et al., 2017). Under-ice blooms may become more common in a scenario of thinner sea ice and a changing under-ice light climate (see also Arrigo et al., 2012). The clear dominance of photoautotrophs >3 μm in the advected bloom (Station1, Table 1) is similar to previous reports from Svalbard waters (Hodal et al., 2011; Marquardt et al., 2016). The OTU assigned to the cold water dinoflagellate *Woloszynskia halophila*, a bloomforming species of the Baltic Sea (Kremp et al., 2005), was highly abundant in the DNA library from the surface (0 m), while very few reads were found in the corresponding RNA library. As 18S sequence divergence is known to not always distinguish species of Dinophyceae (Logares et al., 2007), it is possible that the identified OTU represents another Suessiales species. Interestingly, Suessiales cysts (identified to *Polarella glacialis*; Johnsen et al., submitted) were found in sea ice cores as well as in the pelagic, and this OTU may represent cysts of a Suessiales taxon which is not metabolically active and thus not well represented in the RNA library.

Other potentially photosynthesizing plankton among the abundant OTUs include oligotrich ciliates (Table 2; Figure 5) where several representatives have sequestered chloroplasts that are retained and function as kleptoplastids, enabling photosynthesis by the host (reviewed by Esteban et al., 2010). In support, the very abundant Oligotrichia OTU had particularly high RNA read abundances at 0 and 10 m depth at Station 1, suggesting high metabolic activity of this taxon within the photic zone at this station. However, other Strombidiidae are heterotrophic, and we cannot exclude the possibility that the abundant oligotrichs are bacterivorous as suggested for the abundant *Strombidinium* in the Amundsen Gulf (Terrado et al., 2011).

The community composition of Station 2 was distinctly different from the advected bloom community identified in Station 1 (Figures 4, 6), and although most of the abundant OTUs were present at both stations their relative abundances differed. The arctic clade of *Micromonas* sp. (Arctic *Micromonas*) recruited over 30% of the reads in both RNA and DNA libraries from PW in Station 2, in line with this cold-and shade adapted ecotype of *Micromonas* (Lovejoy et al., 2007) being important in blooms developing in colder water (see also Metfies et al., 2016). In a recent temporal study from Isfjorden, West Spitsbergen, Arctic *Micromonas* had especially high relative read abundances during pre- and post-bloom stages (Marquardt et al., 2016), in agreement with Station 2 being in a post-bloom (alternatively pre-bloom) stage. The dominance of Arctic *Micromonas* over *Phaeocystis* sp. in the colder station 2 suggests that development in this area, located further to the northeast, was less influenced by the large pelagic bloom advected under the ice at Station 1.

An OTU assigned to the cryptomonad *Teleaulax gracilis* was abundant in surface waters of both stations, supporting the developed stages of the blooms as cryptophytes often appear late in the bloom (Leu et al., 2006). RNA read counts suggested that the cryptomonad was especially active at Station 1, in agreement with the ongoing bloom. Fluorescence and PAR were the two environmental parameters identified to separate the two surface communities (Figure 4), suggesting that succession and light conditions were the main drivers of the community differentiation in the surface.

The samples collected around the halocline (45 and 65 m in Station 1) had increased abundances of OTUs assigned to the autotrophs *Phaeocystis* sp. and *T. antarctica* probably reflecting sinking of the huge bloom (Figure 5). The same pattern was seen for an OTU assigned to another bloom species, *Chaetoceros socialis* (data not shown), which also was abundant in microscopy counts from the vicinity (Johnsen et al., submitted). Abundant heterotrophic flagellates most likely feeding on the sinking bloom included the ciliates Choreotrichia-1 and Protostomatea, Picozoa, and MAST 7 (ANT12_10; Figure 5). MAST 7 is more often found in and below the subsurface chlorophyll maximum layer compared to the upper mixed layer in the Arctic Ocean (Monier et al., 2013).

As commonly found in studies of picoplankton diversity based on filtration approaches, sequences from larger protists and metazoans were recovered, probably due to cell breakage and deformation of flexible walled cells allowing their DNA and RNA to pass through the 3 μm filters (Terrado et al., 2011; Sørensen et al., 2013). Another technical issue using 18S sequence data to compare protist communities is the potential for overestimation of certain OTUs due to high rDNA copy numbers or artifacts of the sequencing procedure (e.g., Zhu et al., 2005; Potvin and Lovejoy, 2009).

### Atlantic water communities are highly diverse and similar despite geographical distance

The increased diversity and evenness found in the AW microbial communities compared to the PW surface samples (Table 1), suggest that these deep communities are both diverse and metabolically active. Also, the increased representation of rare taxa in AW libraries may represent a seeding stock that could function as a biological buffer to environmental change (Sogin et al., 2006; Pedros-Alio, 2012). The deep, dark and nutrient-rich Atlantic current extends from the Norwegian Coastal Current, flowing in a 200–500 m deep layer. This deep AW layer may access the sunlit part by upwelling processes, as was shown to occur in January 2012 onto the shelf of northern Spitsbergen (Falk-Petersen et al., 2015). The AW harbors a distinct community of microbial protists brought northwards with the North Atlantic Current from more southern waters as well as Arctic species introduced through mixing with local water. The increased temperature identified in the waters of outer Isfjorden over the last decade (Pavlov et al., 2013) suggests that the organisms transported northwards with the West Spitsbergen Current may have increased survival, both in the deep and if they are transported to the surface. As argued by Pedros-Alio (2012), the rare biosphere may be composed of species whose individual requirements do not fit the current environment, but that may be able to bloom under different conditions.

There were distinct differences in the abundances of several OTUs occurring in the deeper AW between the DNA and RNA libraries (Figure 5). Most of the OTUs predominating in the AW (MALV, RAD, Labyrinthulaceae, Mesodiniidae) have heterotrophic or parasitic lifestyles, except the Mesodiinidae which have representatives at different trophic levels. Radiolarians are known to be associated with a large diversity of marine alveolates of which they are often hosts (Guillou et al., 2008; Bråte et al., 2012). The radiolarian OTU RAD B (Taxopodida; Suzuki and Not, 2015) and the five most abundant marine alveolate OTUs (Figure 5) were all abundant in the AW DNA libraries, but were not active (i.e., not abundant in the RNA libraries), as has previously been shown in other studies (Not et al., 2009; Hu et al., 2016; Marquardt et al., 2016). The limited activities shown by the parasitic protists (RAB B and MALV) in AW suggests that these taxa occur in less active life cycle forms.

Radiolaria are known to host a wide diversity of eukaryote symbionts, and were shown to harbor both the MALV I and MALV II lineages (Bråte et al., 2012). Whether the association between RAD B and MALV lineages in the deep AW is due to a parasite-host relationship is unknown, but a similar correlation of MALV sequence abundances with radiolarians was found also in samples from the Western Antarctic Peninsula with increased abundances in deep waters (Cleary and Durbin, 2016).

Other OTUs displayed depth or station related patterns in their RNA to DNA ratios, suggesting that their activity levels were modulated by changes in the environment. One such case was the very abundant dinoflagellate Dino_Clone_NIF_3A1 that only had high activity levels in the deep, suggesting it to be a heterotrophic taxon (Figure 5).

All of the abundant phototrophic OTUs were detected in the AW, albeit at reduced read numbers. Interestingly *Phaeocystis* sp. was also abundant in the AW at Station 1, both in the DNA and RNA libraries, showing that the detected cells were alive. This finding is in agreement with previous reports on viable *Phaeocystis* sp. in the deep (Vader et al., 2014), suggesting that these haptophytes could have a mixotrophic lifestyle as was shown for Arctic *Micromonas* (McKie Krisberg and Sanders, 2014).

In conclusion, the microbial eukaryote communities of the two under-ice stations differed due to a combination of their water mass history and local processes, the homogenizing effect of water currents being less important in surface waters. Furthermore, the combined use of RNA and DNA libraries allow interpretation on the activity levels of the identified taxa. Thus, an extended use of RNA libraries in similar studies may improve our understanding of the active part of these communities.

## Accession numbers

The 454 sequencing data obtained in this study was submitted to the Sequence Read Archive (SRA) at NCBI GenBank (BioProject ID PRJNA384116).

## Author contributions

AM, AV, and TG participated during the field sampling. AM and AV did the molecular lab work, and SK analyzed the samples for nutrient concentrations. AM did the bioinformatic analyses with input from AV and TG. AM drafted the manuscript text, and all co-authors contributed to discussing the results and editing the manuscript.

### Conflict of interest statement

The authors declare that the research was conducted in the absence of any commercial or financial relationships that could be construed as a potential conflict of interest.
