# Supplementary material for: Microbial Eukaryotes in an Arctic Under-Ice Spring Bloom North of Svalbard
Source: Front Microbiol. 2017 Jun 28;8:1099. doi: 10.3389/fmicb.2017.01099 (PMC5487457; doi:10.3389/fmicb.2017.01099)
Supplement: Supplementary file 1 [file Table1.docx]

Supplementary Information (SI)

**Microbial Eukaryotes in an Arctic Under-Ice Spring Bloom North of Svalbard**

Archana R. Meshram, Vader A, Kristiansen S, Gabrielsen TM

**Figure S1.** Rarefaction curves of DNA and RNA sequences at 98% clustering threshold after excluding the singletons and computed every 100 sequences using 5000 subsampling iterations. The naming of the samples are as follows: 1 and 2 represent the station number, D and R represents the DNA/RNA sample, followed by the depths of the respective station (e.g.1D0 - is a DNA sample of 0 m from Station 1).

**Figure S1.**
